# A novel deep learning-driven framework for improving lncRNA comprehensive annotation with LncADeep 2.0

**DOI:** 10.1093/bioinformatics/btag162

**Published:** 2026-04-01

**Authors:** Yiyan Zhou, Jiaheng Hou, Haoling Xie, Nuoshi Lin, Cheng Yang, Hengchuang Yin, Wanqiu Ding, Huaiqiu Zhu

**Affiliations:** Department of Biomedical Engineering, College of Future Technology, Peking University, Beijing 100871, China; Department of Big Data and Biomedical AI, College of Future Technology, Peking University, Beijing 100871, China; Center for Quantitative Biology, Academy for Advanced Interdisciplinary Studies, Peking University, Beijing 100871, China; Department of Biomedical Engineering, College of Future Technology, Peking University, Beijing 100871, China; Department of Big Data and Biomedical AI, College of Future Technology, Peking University, Beijing 100871, China; Center for Quantitative Biology, Academy for Advanced Interdisciplinary Studies, Peking University, Beijing 100871, China; College of Life Sciences, Beijing Normal University, Beijing 100875, China; Department of Biomedical Engineering, College of Future Technology, Peking University, Beijing 100871, China; Department of Big Data and Biomedical AI, College of Future Technology, Peking University, Beijing 100871, China; Center for Quantitative Biology, Academy for Advanced Interdisciplinary Studies, Peking University, Beijing 100871, China; Department of Biomedical Engineering, College of Future Technology, Peking University, Beijing 100871, China; Department of Big Data and Biomedical AI, College of Future Technology, Peking University, Beijing 100871, China; Center for Quantitative Biology, Academy for Advanced Interdisciplinary Studies, Peking University, Beijing 100871, China; Department of Biomedical Engineering, College of Future Technology, Peking University, Beijing 100871, China; Department of Big Data and Biomedical AI, College of Future Technology, Peking University, Beijing 100871, China; Center for Quantitative Biology, Academy for Advanced Interdisciplinary Studies, Peking University, Beijing 100871, China; Department of Big Data and Biomedical AI, College of Future Technology, Peking University, Beijing 100871, China; Department of Biomedical Engineering, College of Future Technology, Peking University, Beijing 100871, China; Department of Big Data and Biomedical AI, College of Future Technology, Peking University, Beijing 100871, China; Center for Quantitative Biology, Academy for Advanced Interdisciplinary Studies, Peking University, Beijing 100871, China

## Abstract

**Motivation:**

Long non-coding RNAs (lncRNAs) have emerged as crucial players in diverse physiological and pathological processes, yet the biological mechanisms of the vast majority of lncRNAs remain elusive. To fill this gap, it is necessary to improve the accuracy of lncRNA identification and functional annotation.

**Results:**

Here, we introduce LncADeep 2.0, an integrated deep learning framework designed to meet these needs. In the identification module, LncADeep 2.0 incorporated novel peptide features along with sequence and structural information, demonstrating superior performance over our previous LncADeep and other existing tools on both annotated transcripts from GENCODE and RNA-seq data. For functional annotation, LncADeep 2.0 leveraged lncRNA-centric interaction networks and gene ontology terms through the transfer learning strategy to achieve robust annotation performance with limited functional data. Compared to LncADeep, LncADeep 2.0 could accurately elucidate the general functions of given lncRNA sequences, predict tissue- or cell-type-specific functions from bulk and single-cell RNA-seq data, and establish connections between tumor-associated lncRNAs and genomic markers. Overall, LncADeep 2.0 stands out as an efficient and reliable tool for lncRNA identification and functional annotation across a wide spectrum of biological processes.

**Availability and implementation:**

LncADeep 2.0 is available for use at https://github.com/Jefferson-Chou/LncADeep2 and https://doi.org/10.5281/zenodo.17164767.

## 1 Introduction

The human genome is predominantly transcribed, with the majority of transcripts being non-coding RNAs that are not translated into proteins. Among them, lncRNAs are defined as transcripts that exceed 200 nucleotides in length and have emerged as pivotal players in a wide variety of biological processes (Mattick *et al.* 2023) and pathological conditions, such as cancers (Huarte 2015) and autoimmune diseases (Wu *et al.* 2015). Notably, lncRNAs exhibit tissue-specific (de Goede *et al.* 2021) and cell-type-specific (Mattick *et al.* 2023) expression patterns, enabling precise functional regulation in spatiotemporal biological contexts.

However, the majority of lncRNAs remain uncharacterized, with many requiring further functional annotations, especially under specific biological conditions. In the past decades, the rapid advancement of high-throughput sequencing technologies has generated an unprecedented volume of transcriptomic data, facilitating the systematic identification of novel lncRNAs and elucidation of their functions.

A variety of tools have been developed to discriminate lncRNA transcripts from mRNAs. As for the features utilized, these models primarily rely on sequence intrinsic features like Fickett score, open reading frame (ORF) length, ORF integrity and k-mer frequency. Tools like LncDC (Li and Liang 2022) and LncFinder (Han *et al.* 2019) also take account of the secondary structure features. Meanwhile, physicochemical property of proteins or electron–ion interaction pseudo-potential is proved to be effective (Kang *et al.* 2017, Tong and Liu 2019, Li and Liang 2022). As for the classifiers, the majority of models are implemented by conventional machine learning algorithms like XGBoost and SVM (Sun *et al.* 2013, Kang *et al.* 2017, Han *et al.* 2019, Tong and Liu 2019, Li and Liang 2022), while some tools typically employ deep learning models, such as the convolutional neural network, deep stacking network and deep belief network (Fan and Zhang 2015, Yang *et al.* 2018, Li *et al.* 2024). Generally speaking, many existing tools exhibit limitations in fully utilizing abundant features or fail to harness the full potential of advanced deep learning models.

After identification, deciphering the functions of lncRNAs poses a more critical and challenging task. The “guilt by association” approach via statistical tests (Jiang *et al.* 2015) or heterogenous global networks (Guo *et al.* 2013, Spetale *et al.* 2021, Zhang *et al.* 2021) is a commonly employed strategy to predict co-expressed or correlated genes of lncRNAs, based on which, enrichment analyses (Ke *et al.* 2020) or machine learning methods (Spetale *et al.* 2021, Deng *et al.* 2023) can be applied for finding gene ontology (GO) terms to elucidate lncRNA functions. Nevertheless, these approaches obviously suffer from the insufficiency of data that links lncRNAs to GO terms, increasing the risk of model overfitting. Meanwhile, the lack of data exacerbates lncRNAs functions prediction as some of these tools are limited to the GO terms available within the training set, which poses a significant obstacle to the exploration of other latent functions. Considering the specific expression patterns of lncRNAs, leveraging RNA‑seq data derived from specific biological conditions offers great potential to enhance the functional annotation of lncRNAs. However, most existing tools fail to fully exploit RNA‑seq data to characterize the tissue‑ and cell‑type‑specific functions of lncRNAs. Addressing the above challenges requires an integrated computational pipeline capable of *de novo* identification and functional annotation. Now, driven by the exponential growth of sequencing data and major algorithmic advances, we introduce LncADeep 2.0, an end-to-end workflow for lncRNA identification and functional annotation. In its identification module, LncADeep 2.0 integrates peptide features into deep learning networks, demonstrating superior performance over LncADeep (Yang *et al.* 2018) and other state-of-the-art tools on both annotated transcripts from GENCODE and RNA-seq data. For functional annotation, the module employs transfer learning by leveraging lncRNA-centric interaction networks to establish robust links between lncRNAs and GO terms. By further integrating expression profiles, LncADeep 2.0 significantly enhances tissue- and cell-type-specific functional insights. Case studies demonstrate that our framework can successfully infer novel GO annotations for lncRNAs on the basis of known functions and propose new associations between lncRNAs and established tumor genomic markers.

## 2 Materials and methods

### 2.1 Overview of LncADeep 2.0

**Figure 1:**
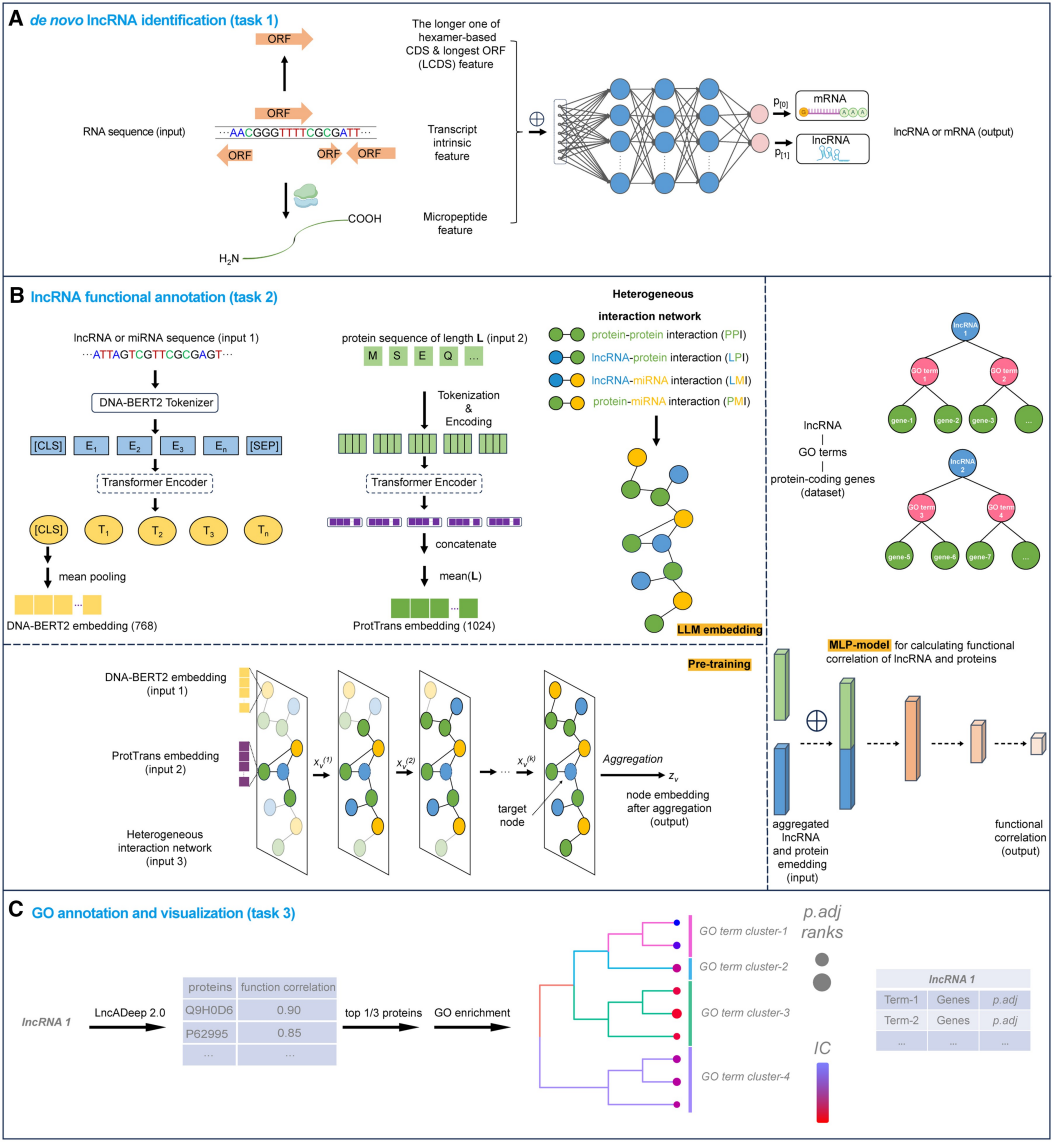
Workflow of LncADeep 2.0. (A) Task 1 of LncADeep 2.0 for *de novo* lncRNA identification. An MLP model incorporating three sources of features is utilized to distinguish lncRNA transcripts from mRNA transcripts using RNA sequences as inputs. (B) Task 2 of LncADeep 2.0 for lncRNA functional annotation. A transfer learning strategy is leveraged by integrating three subsections: large language model embedding, pre-training via a graph neural network and training an MLP model. (C) Task 3: GO annotation and visualization of predicted function-related proteins. The outputs for each lncRNA include the enriched GO terms and the corresponding clustered tree plot. The Figure 1A was created with Biorender software.

LncADeep 2.0 comprised three modules: (i) the *de novo* lncRNA identification module (task 1) (Fig. 1A), (ii) the lncRNA functional annotation module (task 2) (Fig. 1B), and (iii) the GO annotation and visualization module (task 3) (Fig. 1C). In the identification module of LncADeep 2.0, we introduced several novel features for embedding lncRNA and mRNA sequences compared with LncADeep. A multilayer perceptron (MLP) model was used for classification of lncRNA or mRNA. For the functional annotation of lncRNAs, we leveraged a transfer learning strategy to substantially reduce the limitations of the insufficiency of lncRNA functional datasets. The source task for the transfer learning involved predicting direct and two-hop interacting proteins of lncRNAs. Leveraging the knowledge gained from the source task, the target task focused on identifying function-related proteins of lncRNAs. The pre-trained model designed for the source task was a heterogeneous graph neural network, integrating the interactions between protein–protein, lncRNA–protein, lncRNA–miRNA, and protein–miRNA. Features extracted by the graph neural network were subsequently employed in the target task of predicting function-related proteins of lncRNAs, through utilizing an MLP as the predictive model. GO enrichment analyses was then performed on the predicted proteins for each input lncRNA to decipher the potential functions of lncRNA.

### 2.2 Data preprocessing

In the identification module, sequences of human lncRNA and mRNA transcripts for five-fold cross-validation were downloaded from GENCODE (Frankish *et al.* 2019) version 45. To keep only representative sequences, redundancy removal of lncRNA and mRNA sequences was performed using CD-HIT (Fu *et al.* 2012) (Table 1, available as supplementary data at *Bioinformatics* online), keeping 58 736 lncRNAs and 108 647 mRNAs.

In the functional annotation module, we developed a transfer learning strategy. For the source task, we constructed the human lncRNA interacting network comprising three types of nodes and four types of edges (Table 1). In this network, lncRNA–protein interaction (LPI) and lncRNA–miRNA interactions (LMIs) were obtained from starBase (Li *et al.* 2014), protein–miRNA interactions (PMIs) were sourced from RNAInter database (Kang *et al.* 2022), protein–protein interactions (PPIs) were retrieved from STRING database v12 (Szklarczyk *et al.* 2023). Data cleaning was performed to retain molecules with accessible sequences and standardized identifiers. Specifically, lncRNAs were assigned Ensembl IDs, proteins were assigned UniProt IDs, and miRNAs were assigned miRBase IDs.

**Table 1:** Human lncRNA-centric interacting network dataset after cleaning.

Interacting pairs	Number of interactions	Number of molecules included
LPI	167 392	281 proteins and 12 624 lncRNAs
LMI	30 658	641 miRNAs and 1258 lncRNAs
PMI	97 248	9619 proteins and 641 miRNAs
PPI	13 252 986	19 029 proteins

The target task was to predict function-related proteins of lncRNAs. To this end, 110 human lncRNAs and 21 GO terms (biological processes, BP), including 1724 proteins, were selected for training and testing (Supplementary Methods, available as supplementary data at *Bioinformatics* online).

In order to compare the performance of GO annotation prediction tools for lncRNAs, a manually curated dataset from Deng *et al.* (2023), lncRNA2GO-68 dataset, was used for evaluation. The dataset comprised 69 human lncRNAs with 72 GO annotations covering three ontologies (B.P., C.C., and M.F.). For our assessment, we selected the BP-related pairs which included 67 lncRNAs and 46 GO terms.

### 2.3 Feature selection and model construction for identification

In the identification module, mRNA and lncRNA sequences contained three kinds of features: features of the longest CDS (LCDS), intrinsic transcript features, and peptide features. Among these, LCDS features and part of intrinsic transcript features were inherited from LncADeep.

An ablation experiment on the novel features was conducted. These novel features included ORF integrity (a Boolean value telling if the ORF of the transcript was complete) and five representative physicochemical features of peptides including instability index, isoelectric point (pI), grand average of hydropathicity (GRAVY), molecular weight (Mw), and the combination of pI and Mw (pI_Mw = log10(Mw/pI + 1)). Besides, DNABERT2 (Zhou *et al.* 2024) pre-trained model was also used for testing whether it was suitable for transcripts embeddings.

We designed an MLP model for classification of lncRNA and mRNA. The dimensions of three hidden layers were 128, 64, and 32. In the ablation experiment, the input layer dimension ranged depending on the input feature.

### 2.4 Model architecture for transfer learning and annotation visualization

The pre-training model designed for the source task was a heterogeneous graph neural network, including three primary components: node embedding transformation layers, a GraphSAGE-based graph neural network (Hamilton *et al.* 2017) module, and an edge classification module. The interaction network graph (G) comprised three distinct types of nodes (V) and four types of edges (E): G=(V,E). For embedding the lncRNA and miRNA nodes, the DNABERT2 model was utilized, while the ProtTrans (Elnaggar *et al.* 2022) model was employed for embedding protein nodes. For each node type, separate linear transformation layers were implemented to project original feature of each node type into a unified feature ${\text{h}}_{m}^{0}$, which was given as


(1) $$h_{m}^{\left(0\right)}=W_{m}x_{m}(m\in \{\text{lncRNA},\;\text{miRNA},\;\text{protein}\}),$$

herein, x represented the initial features of the nodes, and W was the trainable weight matrices.

At the core of the model lay a three-layer GraphSAGE convolutional neural network designed to capture complex interaction patterns within the heterogeneous network. Each GraphSAGE layer maintained the same dimensionality (150) for both input and output features. The message passing function was formulated as:


(2) $$h_{A}^{\left(l\right)}=\text{ReLU}(W_{A}^{\left(l\right)}h_{A}^{\left(l-1\right)}+\sum_{B\in N\left(A\right)}\frac{1}{\left|N\left(A\right)\right|}W_{A\leftarrow B}^{\left(l\right)}h_{B}^{\left(l-1\right)}).$$

Here, A, B represented any node types among lncRNA, miRNA, and protein, respectively. $h_{A}^{\left(l\right)}$ was the output of the *l*th layer for node type A. $W_{A}^{\left(l\right)}$ and $W_{A\leftarrow B}^{\left(l\right)}$ were the related weight matrices. N(A) denoted the set of neighboring node types connected to node type A.

After the graph neural network, the refined node embeddings were utilized to predict interactions between lncRNAs and proteins. The edge classification module comprised three fully connected layers, enabling the model to discern the presence and type (direct or 2-hop) of interactions between lncRNAs and proteins based on their embedded feature representations, which could be expressed as:


(3) $$\begin{array}{c} y_{\left(i,j\right)}=W_{3}\cdot \text{ReLU}\left(W_{2}\cdot \text{ReLU}\left(W_{1}\cdot \text{Concat}\left(h_{i},h_{j}\right)\right)\right). \end{array}$$

Here, **y**_(__*i*__,_*_j_*_)_ was the classification result for the edge between node *i* and node *j*. **h**_*i*_ and **h**_*j*_ were the learned features of node *i* and node *j* from the previous graph neural network. $W_{1}$, $W_{2}$  $W_{3}$ were the weight matrices of the classifier’s linear layers. The rectified linear unit (ReLU) is a widely adopted activation function in deep learning, defined as f(x)=max(0,x).

Features extracted by the graph neural network were subsequently employed in the target task of predicting function-related proteins of lncRNAs. To perform this prediction, we utilized an MLP as the predictive model. GO enrichment analysis was then performed on the predicted proteins for each input lncRNA using the R package *clusterProfiler* (Wu *et al.* 2021) and visualized with appropriate parameters (Supplementary Methods, available as supplementary data at *Bioinformatics* online).

### 2.5 Evaluation of LncADeep 2.0 performance across available tools

In order to compare the performance of lncRNA identification for human transcripts from GENCODE, a five-fold test are conducted. LncRNA transcripts were as positives and mRNA transcripts as negatives to calculate the following metrics: area under the receiver operating characteristic curve (AUROC), precision, recall, and *F*1-score. They were defined as follows:


(4) $$\text{Precision}=\frac{\text{TP}}{\text{TP}+\text{FP}},\text{Recall}=\frac{\text{TP}}{\text{TP}+\text{FN}},F1-\text{score}=\frac{2*\text{Precision}*\text{Recall}}{\text{Precision}+\text{Recall}}.$$

where TP, TN, FP, and FN were, respectively, the numbers of true positives, true negatives, false positives, and false negatives in the test sets.

Furthermore, to evaluate the performance of various tools in differentiating transcripts obtained from RNA-seq data, we retrieved 5702 lncRNA-labeled transcripts and 121 184 mRNA-labeled transcripts from RNA-seq data of human hypertrophic cardiomyopathy (Liu *et al.* 2019). These labeled transcripts were used as the independent test set for measurement.

To evaluate the functional annotation module, we performed three aspects of assessments: (i) the performance of predicting function-related proteins of lncRNAs using functional annotation evaluation dataset A; (ii) the examination of potential negative transfer; and (iii) the performance of GO annotation prediction using functional annotation evaluation dataset B (Supplementary Methods, available as supplementary data at *Bioinformatics* online).

### 2.6 Prediction of lncRNAs’ tumor association and functions for RNA-seq data

To predict lncRNAs’ tumor association, the tumor genomic markers were retrieved from two sources: the top 20 tumor genomic markers in the database COSMIC (Sondka *et al.* 2024) as well as cancer risk genes from the database OMIM (Amberger *et al.* 2019). LncRNAs associated with eight types of tumors (bladder cancer, breast cancer, colorectal cancer, gastric cancer, hepatocellular carcinoma, lung cancer, prostate cancer, and renal cell carcinoma) were retrieved from the database Lnc2Cancer 3.0 (Gao *et al.* 2021) whose items were supported by experimental researches.

LncRNAs belonging to the tumor-unrelated group (not included in the Lnc2Cancer database) and having a predicted marker count of 15 or more (equivalent to three fourths of the total 20 tumor genomic markers obtained from the COSMIC database) were selected for survival analyses.

To annotate the functions of tissue-specifically expressed lncRNAs, bulk RNA-seq data from the brain cortex, spleen, and testis were obtained from the GTEx Analysis V10 release (Coorens *et al.* 2025). Gene TPMs were then utilized for downstream analyses. For each tissue, tissue-specifically expressed lncRNAs were defined as those meeting two criteria: (i) TPM > 1 in the target tissue (ensuring baseline expression); (ii) fold change >4 compared to the average TPM of other tissues (indicating strong tissue-specificity).

For the functional annotation of metastasis associated lung adenocarcinoma transcript 1 (*MALAT1*), both bulk RNA-seq data and single-cell RNA sequencing data (scRNA-seq) were used to elucidate the potential functions in tissue- and cell-type-specific conditions. Bulk RNA-seq data of the tissue from breast cancer patient (sample ID “TCGA-BH-A18K-01A-11R-A12D-07”) was based upon data generated by the TCGA Research Network: https://www.cancer.gov/tcga, while the bulk RNA-seq data in the brain cortex were sourced from GTEx. The scRNA-seq data were sourced from two publications: (i) caudal ganglionic eminence (CGE) interneurons were obtained from Chen *et al.*'s brain cell atlas (Chen *et al.* 2024); (ii) malignant epithelial cells associated with lung adenocarcinoma (LUAD) were derived from Wu *et al.*'s profiling of non-small cell lung cancer (NSCLC) cellular heterogeneity (Wu *et al.* 2021).

PCGs expressed based on sequencing data were prioritized for the functional annotations of lncRNAs. Specifically, in the bulk RNA-seq mode, ubiquitously expressed housekeeping genes were first filtered according to the list provided by Eisenberg and Levanon (2013) to ensure the focus on genes with potential tissue-specific pattern. The remaining PCGs were ranked by expression levels, and the top 50% ones were selected. In the scRNA-seq mode, housekeeping genes were also removed while the remaining expressed PCGs were all kept considering the dropout in the scRNA-seq data. PCGs with high relation score were regarded as function-related proteins. The GO enrichment results of these function-related proteins were as the functional annotations for the input lncRNAs.

## 3 Results

### 3.1 LncADeep 2.0 exhibited superior ability in lncRNA identification and functional annotation

Ablation experiments were conducted to evaluate the impact of the novel features introduced in LncADeep 2.0. The results demonstrated that ORF integrity of the transcript, isoelectric point (pI), grand average of hydropathicity (GRAVY), molecular weight (Mw), and the combination of pI and Mw (pI_Mw = log10(Mw/pI + 1)) of peptides could significantly facilitate the identification task (Table 2, available as supplementary data at *Bioinformatics* online).

**Figure 2:**
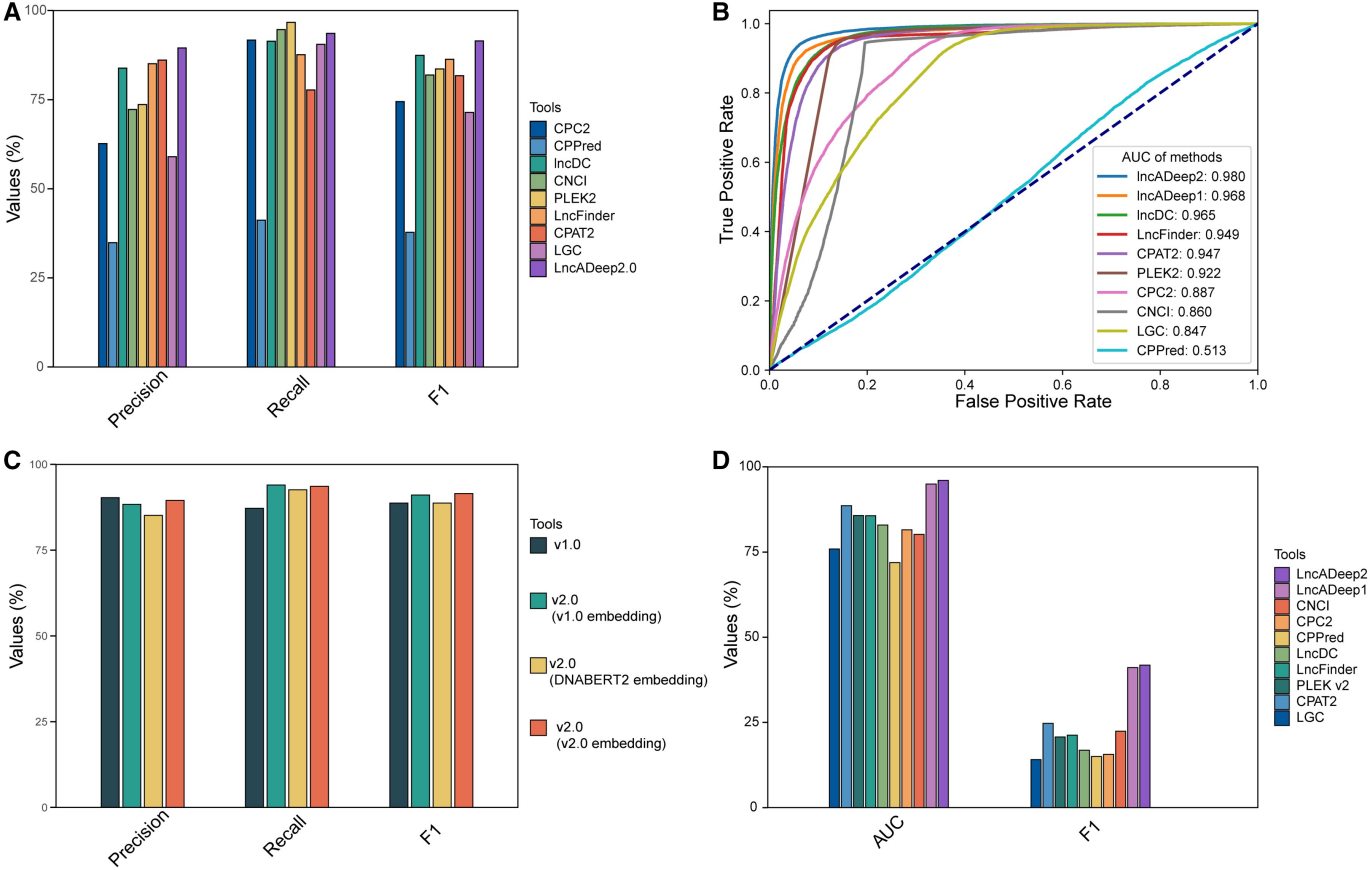
Performance of LncADeep 2.0 and other tools on lncRNA identification. (A) Five-fold comparison on the annotated transcripts from GENCODE. (B) ROC curves of different tools. (C) Comparison between LncADeep 2.0 and version 1.0. (D) Evaluation on an independent bulk RNA-seq test set (5702 lncRNAs and 121 184 mRNAs).

The ability of classifying lncRNA and mRNA transcripts was further tested on human transcripts from GENCODE (v45). LncADeep 2.0 was compared with LncADeep as well as eight additional lncRNA identification tools: LncDC (Li and Liang 2022), PLEK v2 (Li *et al.* 2024), CPC2 (Kang *et al.* 2017), CNCI (Sun *et al.* 2013), CPPred (Tong and Liu 2019), CPAT2 (Wang *et al.* 2013), LGC (Wang *et al.* 2019), and LncFinder (Han *et al.* 2019). Among the above tools, LncDC, CPAT2, and LncFinder were retrained by their model-training options. For the tools without available retraining option, namely CPC2, CNCI, LncADeep, CPPred, LGC, and PLEK v2, we used their pre-built models. The results showed that LncADeep 2.0 outperformed the existing tools, with the highest *F*1-score 0.915 and AUROC 0.980 (Fig. 2A and B). When employing the large language model DNABERT2 for embedding, the results indicated that the features of DNABERT2 were less effective than the novel features utilized in our model (Fig. 2C).

In addition, lncRNA and protein-coding transcripts obtained from RNA-seq data were employed as an independent test for further comparison. The independent test result also proved the highest *F*1-score and AUC of LncADeep 2.0 (Fig. 2D).

**Figure 3:**
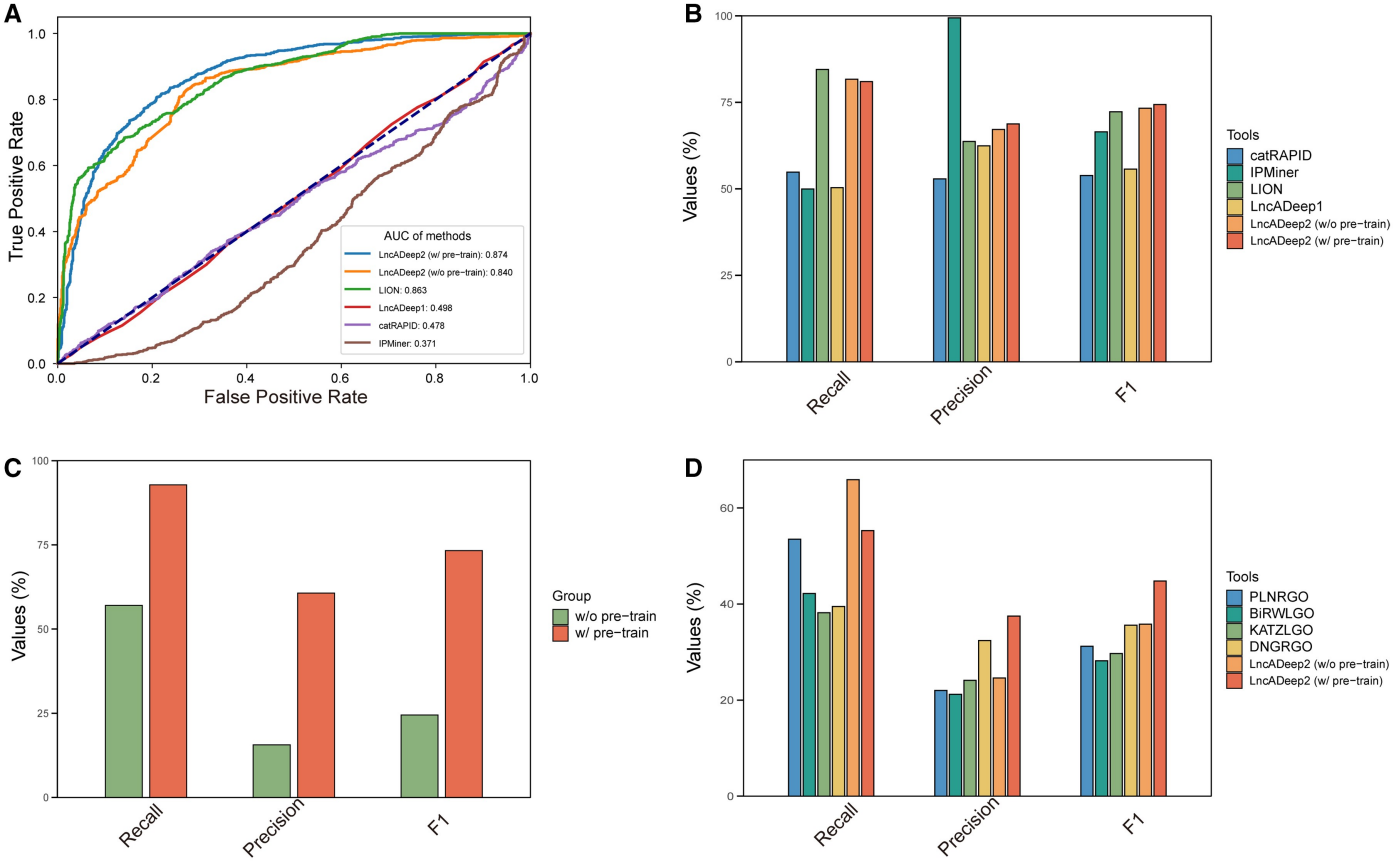
Evaluation of the LncADeep 2.0 functional annotation module. ROC curves (A) and comparisons (B) of LncADeep 2.0 and other tools on lncRNA–protein interaction prediction. (C) Evaluation of transfer learning by comparing pre-trained (w/pre-train) and non-pre-trained embedding (w/o pre-train). (D) GO term prediction performance across tools.

Except for lncRNA identification, LncADeep 2.0 also exhibited superior ability in functional annotation. Using functional annotation evaluation data A (Materials and methods), we compared both the pre-trained and non-pre-trained versions of LncADeep 2.0 with other lncRNA–protein interaction prediction tools, including LION (Han *et al.* 2022), LncADeep, IPMiner (Pan *et al.* 2016), and catRAPID (Armaos *et al.* 2021) (Fig. 3A and B). In this analysis, the non-pre-trained version of LncADeep 2.0 referred to the model in which lncRNA and protein features were derived from DNABERT2 and ProtTrans embeddings, respectively. For both the pre-trained and non-pre-trained versions of LncADeep 2.0, training was halted when the training loss did not decrease for five epochs and the models corresponding to the lowest training loss were picked for comparison. The LION model was retrained using its training option. From the results, our pre-trained model achieved the highest scores in both the *F*1-score 0.744 and the AUROC 0.874 (Fig. 3A and B). To further evaluate if there existed negative transfer, we tested on the functional annotation evaluation data B and the results showed that the pre-trained model significantly outperformed the non-pre-trained model on all metrics, indicating the absence of negative transfer (Table 3, available as supplementary data at *Bioinformatics* online, and Fig. 3C).

In order to evaluate the accuracy of GO terms predicted by LncADeep 2.0, we used the lncRNA2GO-68 dataset for comparing LncADeep 2.0 with other GO terms prediction tools, including DNGRGO (Deng *et al.* 2023), KATZLGO (Zhang *et al.* 2019), BiRWLGO (Zhang *et al.* 2017), and PLNRGO (Deng *et al.* 2018). In terms of recall, precision, and *F*1-score, LncADeep 2.0 performed the best among these methods (Fig. 3D).

### 3.2 LncADeep 2.0 accurately predicted GO annotations for pre-annotated lncRNAs both within and outside the training set

To further validate the capability of LncADeep 2.0 in annotating lncRNAs, we carried out a case study focusing on four annotated lncRNAs possessing crucial regulatory functions in physiological conditions (*XIST* and *MALAT1* within the training set, *SMILR* and *FIRRE* outside the training set). The visualization module was employed to depict the GO annotations of these lncRNAs, highlighting the adjusted *P*-value and information content (IC), which quantifies the specificity of the functional terms (calculation methodologies are detailed in the Supplementary Methods, available as supplementary data at *Bioinformatics* online).

**Figure 4:**
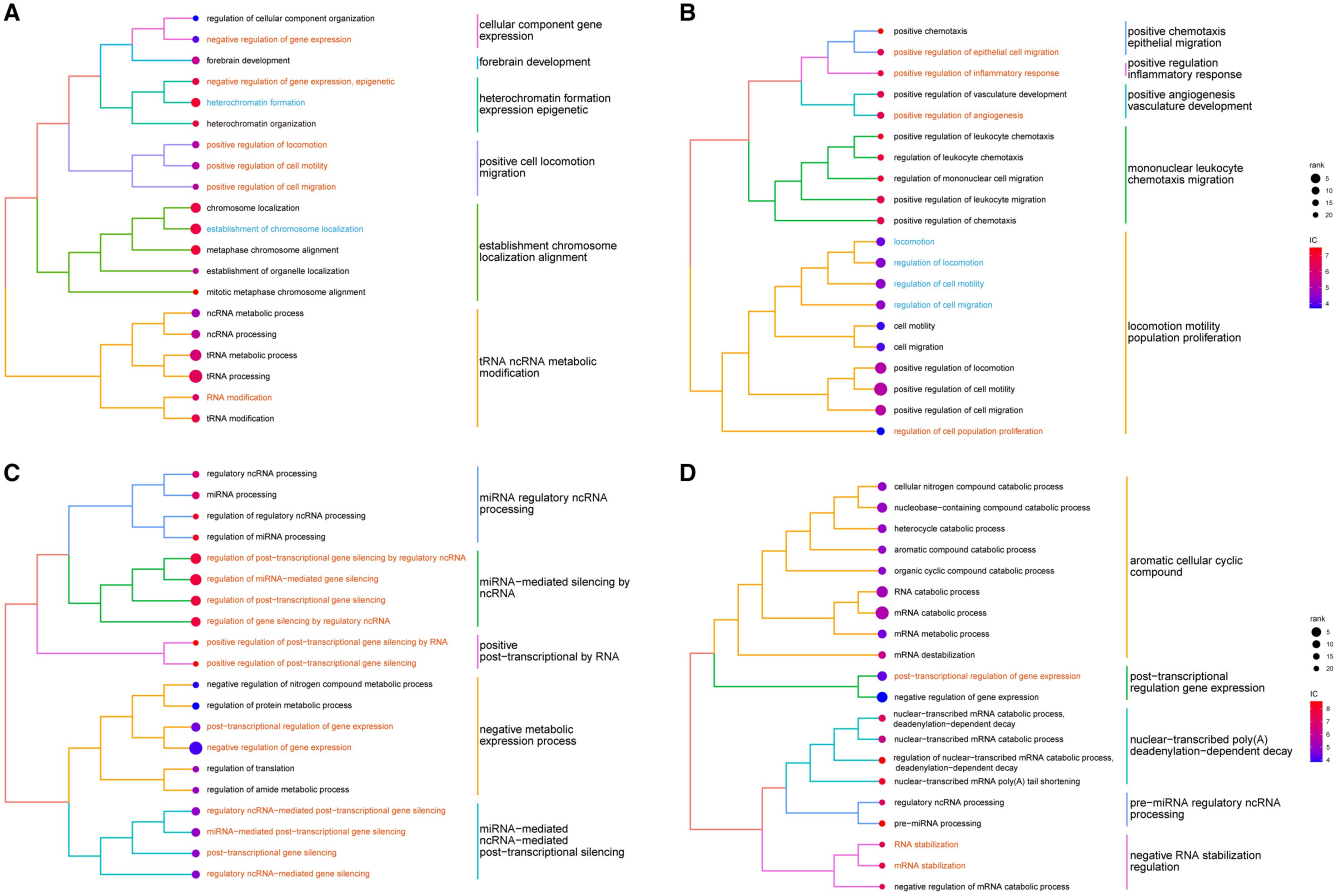
Visualization of functional annotation result for four lncRNAs. GO annotations are shown for lncRNA *XIST* (A), *MALAT1* (B), *SMILR* (C), and *FIRRE* (D). The results are clustered based on similarity and the size of a GO term circle represents the rank of its adjusted *P*-value among all GO terms associated with the lncRNA. Information content (IC) is a metric used to quantify the specificity of a term. GO terms that are more specific have higher IC values. For lncRNAs within the training set, known functions are shown in blue. All other predicted functions validated by literatures are highlighted in orange.

The lncRNA X-inactive specific transcript (*XIST*) acts as a major effector in the X-inactivation process. Notably, LncADeep 2.0 provided precise predictions of functional annotations such as “heterochromatin formation” (GO:0031507) and “negative regulation of gene expression, epigenetic” (GO:0045814). Besides, our annotation for *XIST* (ENSG00000229807) also reported its functions in regulating cell migration and motility (Fig. 4A). This was further corroborated by relevant research that *XIST* promoted cancer cell migration (Naciri *et al.* 2024).

The regulating functions of *MALAT1* (ENSG00000251562) were well-documented and associated with a variety of biological processes (Kim *et al.* 2018, Zhu *et al.* 2023). Its functions in cell migration (GO:0030334) and locomotion (GO:0040012) were also accurately predicted by LncADeep 2.0 (Fig. 4B).

Of note, the lncRNA *SMILR* was absent from the training set. It was reported to negatively regulate gene expression by interacting with miR-141 (Lei *et al.* 2020). In our annotation of *SMILR* (ENSG00000255364), it did have “negative regulation of gene expression” (GO:0010629) and “regulation of miRNA-mediated gene silencing” (GO:0060964), which were precisely predicted (Fig. 4C).

Firre intergenic repeating RNA element (*FIRRE*, ENSG00000213468) was also a lncRNA out of the training set. The term “post-transcriptional regulation of gene expression” (GO:0010608) ranked high in our prediction (Fig. 4D). Accordingly, relevant research findings demonstrate that it modulates the post-transcriptional regulation of inflammatory gene expression by interacting with hnRNPU (Lu *et al.* 2017).

### 3.3 Application of LncADeep 2.0 in predicting the function in tumor development

**Figure 5:**
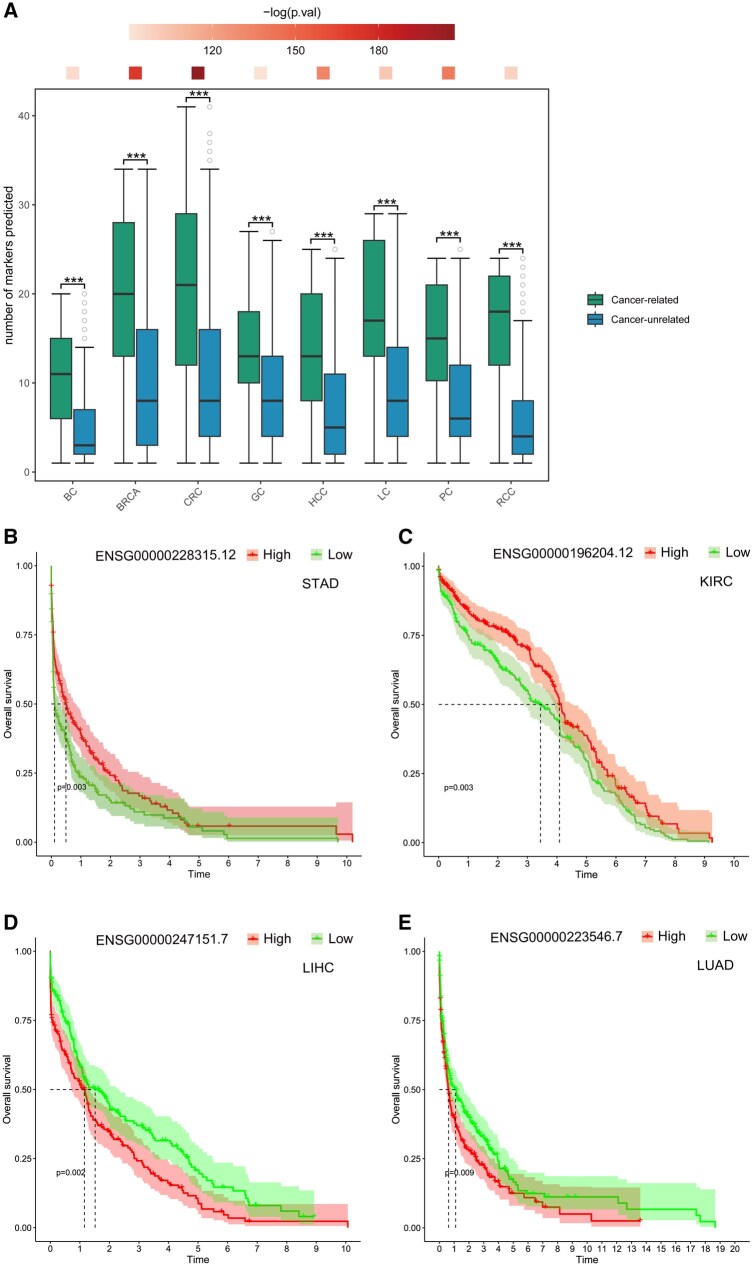
LncADeep 2.0 predicts lncRNAs associated with tumor development. (A) Comparisons of the number of predicted markers associated with lncRNAs in the cancer-related and cancer-unrelated groups (BC, bladder cancer; BRCA, breast cancer; CRC, colorectal cancer; GC, gastric cancer; HCC, hepatocellular carcinoma; LC, lung cancer; PC, prostate cancer; RCC, renal cell carcinoma); two-sided Wilcoxon tests indicated by color bar. (B–E) Survival analysis of stomach adenocarcinoma (STAD), kidney renal clear cell carcinoma (KIRC), liver hepatocellular carcinoma (LIHC), and lung adenocarcinoma (LUAD) patients based on expression levels of the lncRNA *GUSBP11* (ENSG00000228315), *RNF216P1* (ENSG00000196204), *CSTF3-DT* (ENSG00000247151), and *LINC00630* (ENSG00000223546).

To further explore the generalization capabilities of the functional annotation module, we applied LncADeep 2.0 to predict functional correlations between all 12 650 lncRNAs within the interaction network from the pre-training stage and genomic markers sourced from eight distinct types of tumors (Materials and methods) (Figs 3B and 4, available as supplementary data at *Bioinformatics* online). We divided the lncRNAs predictively associated with tumor genomic markers into two groups: the tumor-related group, which comprised tumor-associated lncRNAs supported by evidences in the Lnc2Cancer 3.0 database, and the tumor-unrelated group. We performed a Wilcoxon rank-sum test on the number of predicted markers between these two groups to assess whether LncADeep 2.0 demonstrated efficient functional prediction capabilities for lncRNAs. Across all eight tumor types, our findings revealed that the tumor-related group had a markedly greater number of associated predicted markers compared to the tumor-unrelated group (Fig. 5A).

Moreover, we also discovered that a substantial number of lncRNAs within the tumor-unrelated group exhibited a high degree of correlation with tumor markers (Fig. 3A, available as supplementary data at *Bioinformatics* online). This suggested that these lncRNAs might possess latent functional roles in the process of tumor development. Take breast cancer (BRCA), for example, after reviewing the relevant literature, we found that some lncRNAs within the BRCA-unrelated group could also play vital roles in the development of BRCA (Table 4, available as supplementary data at *Bioinformatics* online). Additionally, to explore whether some of lncRNAs in the tumor-unrelated group could influence tumor patients’ survival, we performed survival analyses. Our results showed that a subset of “tumor-unrelated” lncRNAs significantly impacted tumor patient prognosis (Fig. 5B–E), which were also supported by empirical research findings (Table 5, available as supplementary data at *Bioinformatics* online).

### 3.4 Application of LncADeep 2.0 to predicting lncRNA functions under specific tissues and cell types

**Figure 6:**
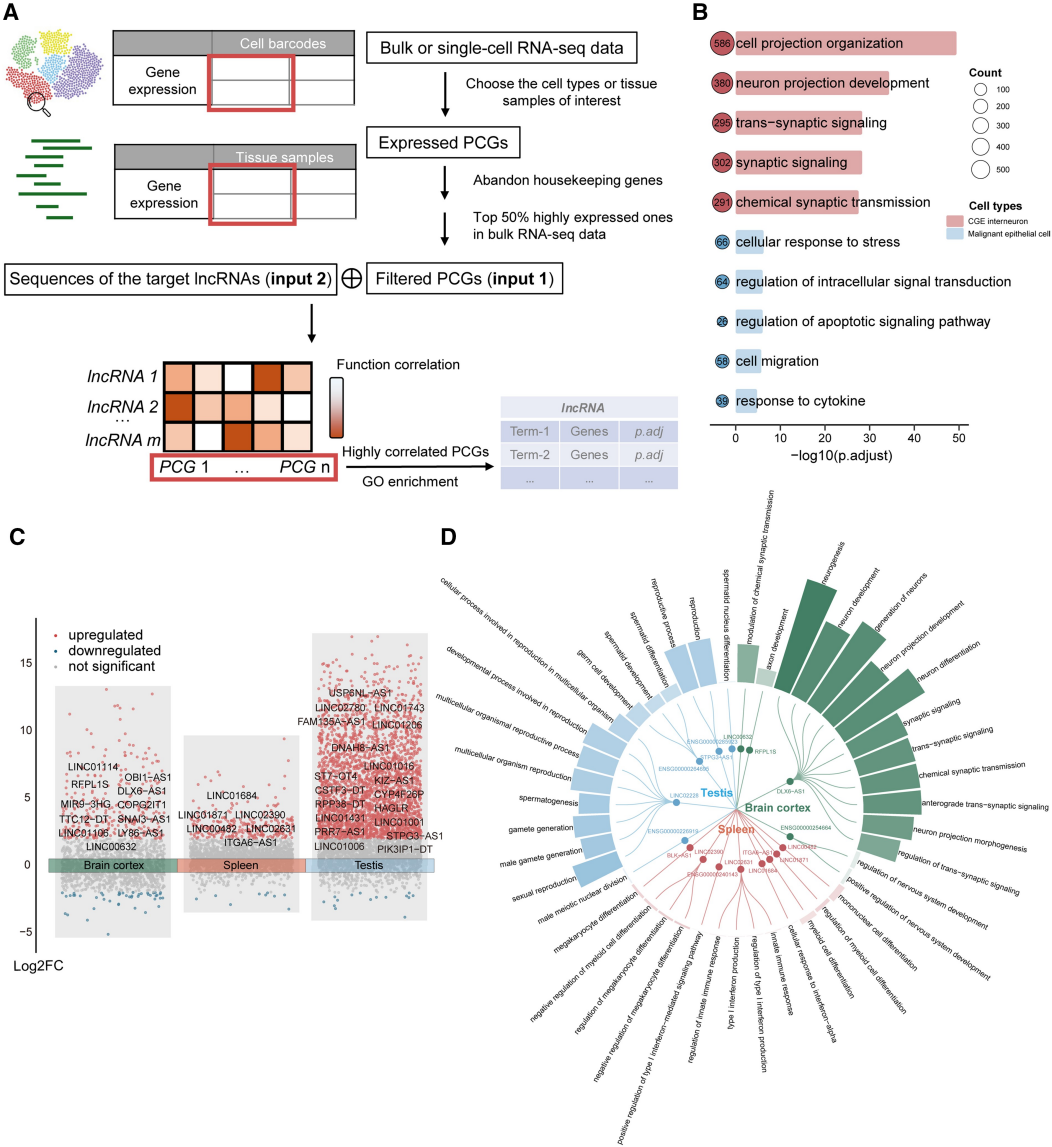
LncADeep 2.0 annotates lncRNA functions under specific cell-type or tissue contexts. (A) The workflow of LncADeep 2.0 for functional annotation using single-cell or bulk RNA-seq data. (B) Functional annotations for *MALAT1* in CGE interneurons from the brain and malignant epithelial cells in NSCLC. (C) LncRNAs showing differential expression patterns (TPM > 1, fold change > 4) across brain cortex, spleen, and testis. Labeled genes are lncRNAs with potential tissue-specific functions. (D) Predicted tissue-specific GO terms for representative lncRNAs in brain cortex, spleen, and testis (top 15 GO terms with the lowest adjusted *P*-values in each tissue).

To assess the ability of LncADeep 2.0 to annotate lncRNA functions under specific cell-type or tissue conditions (Materials and methods and Fig. 6A), scRNA-seq data from the brain and lung cancer were analyzed to predict the functions of *MALAT1* in CGE interneurons from the brain and epithelial malignant cells from NSCLC, respectively (Table 6, available as supplementary data at *Bioinformatics* online, and Fig. 6B).

Furthermore, we conducted functional annotations for lncRNAs that exhibited a tissue-specifically expression pattern in the brain cortex, spleen, or testis (Materials and methods). Specifically, 25, 10, and 106 lncRNAs with GO annotations signifying the potential tissue-specific functions were identified in the brain cortex, spleen, and testis, respectively (Table 6, available as supplementary data at *Bioinformatics* online, and Fig. 6C and D).

Additionally, we intended to predict the function of a given lncRNA within RNA-seq data. We selected bulk RNA-seq samples that displayed the highest expression levels of *MALAT1* in the brain cortex and breast cancer tissues, respectively. Our predictions indicated that the top 20 annotations of *MALAT1* exhibited functions for the specific tissues, which were corroborated by existing literatures. For example, in the RNA-seq data from the brain cortex, we predicted that *MALAT1* correlated with neurogenesis, neuron development, and neuron differentiation (Was *et al.* 2023), as well as synaptic signaling and synapse organization (Bernard *et al.* 2010). In the RNA-seq data from breast cancer, *MALAT1* was predicted to regulate processes like cell migration (Shih *et al.* 2021) and cell population proliferation (Yue *et al.* 2021) (Table 6, available as supplementary data at *Bioinformatics* online).

## 4 Discussion

LncADeep 2.0 is an end-to-end pipeline that can accurately identify and annotate functions of lncRNAs. The identification module was improved with novel features and enriched training data, leading to superior classification performance. Regarding the functional annotation module, unlike most existing tools, which built global co-expression networks to identify related genes and were limited to annotating lncRNAs within the trained network (Zhang *et al*. 2018a, 2018b, Deng *et al.* 2023), our approach adopted a transfer learning strategy and allowed users to analyze any lncRNA based on sequences or expression profiles. Using the GENCODE annotated transcripts and an independent set of RNA-seq data for testing, the benchmarking results indicated that LncADeep 2.0 attained the best *F*1-score, thereby demonstrating the effectiveness of the newly added features in distinguishing mRNA and lncRNA transcripts. For the performance of LncADeep 2.0’s functional annotation module, it surpassed other available tools in predicting both lncRNAs’ function-related proteins and GO terms. The superior performance of the pre-trained model over the non-pre-trained version also demonstrated that it did not lead to negative transfer, thereby indicating the effectiveness of the transfer learning strategy.

LncADeep 2.0 can predict novel functions of known lncRNAs and annotate uncharacterized lncRNAs. For lncRNAs in our training set, like *HOTAIR*, only one function (heterochromatin formation, GO:0031507) is annotated in the training set. Besides predicting entries directly related to “heterochromatin formation”, such as “negative regulation of gene expression, epigenetic,” we can also identify other important functions of *HOTAIR*, e.g. its involvement in central nervous system development (Khani-Habibabadi *et al.* 2022, Deb *et al.* 2024). Except for the item “establishment of chromosome localization” in our dataset, important biological processes that *XIST* is involved in, such as “positive regulation of cell migration,” can also be predicted. With regard to lncRNAs out of the training set, we take *SMIRL* and *FIRRE* as examples, and their roles in the regulation of gene expression can be precisely annotated. In summary, LncADeep 2.0 is a highly generalized model that can predict GO terms matching those in databases as well as novel functions that are supported by the scientific literature.

LncADeep 2.0 is also capable of identifying lncRNAs that potentially perform latent functions during cancer development. The survival analyses suggested that a subset of lncRNAs could significantly affect tumor patient prognosis. Further literature evidence indicated that these lncRNAs could influence tumor development through predicted function-related proteins. These findings demonstrate that our functional prediction algorithm is effective in annotating uncharacterized lncRNAs in diverse physiological and pathological processes.

To further test the generalization capability of LncADeep 2.0 across species, we extended our validation to mouse datasets. Specifically, we tested the identification module on mouse transcripts from the latest GENCODE M38 release. The benchmarking results showed that LncADeep 2.0 achieved the highest performance in distinguishing mouse lncRNAs from mRNAs, reaching an *F*1 score of 0.973 (Table 7, available as supplementary data at *Bioinformatics* online), demonstrating that the designed features and training strategy were not restricted to human transcripts. For the functional annotation module, the predictions for representative mouse lncRNAs showed concordance with curated labels in the Ensembl database (median GO semantic similarity score = 0.71). Altogether, LncADeep 2.0 captures informative sequence and functional features with reasonable cross-species generalizability. Future integration of mouse-specific lncRNA sequence features and interaction networks will be necessary to achieve greater accuracy in both identification and functional annotation.

In addition to elucidating general functions for lncRNAs, LncADeep 2.0 also offers a mode to predict functions under specific tissue or cell-type conditions. We successfully identified lncRNAs with tissue-specific functions in the brain cortex, spleen, and testis. Moreover, for lncRNAs that have been extensively investigated, like *MALAT1*, the tissue-specific annotations we provide are highly consistent with previous literature reports, and we also successfully predict its functions specific to different cell types.

Despite the successful use of transfer learning in LncADeep 2.0 for lncRNA functional annotation, there remain opportunities to further improve the framework across the following areas. First, competitive endogenous RNA (ceRNA) hypothesis represents an important gene regulation mode. In LncADeep 2.0, we take miRNAs, the most crucial molecule in ceRNA networks into account while mRNAs are also indispensable parts of ceRNA networks. Thus, expanding the lncRNA-centric interacting network by adding mRNAs may contribute to the functional annotation of lncRNAs. Second, to address the insufficiency of reliable functional annotations for lncRNAs, we implemented the transfer learning approach, which has been proved to be effective. In both the source task (predicting direct and 2-hop interacting proteins of lncRNAs) and the target task (predicting function-related PCGs of lncRNAs), negative samples were generated by randomly sampling pairs outside the set of known interactions, given the challenges of defining authentic negative samples. However, many of the currently unconfirmed pairs may not actually represent unrelated pairs, which calls for further investigation and experimental validation. Third, we used GO enrichment results as the functional annotation for lncRNAs. Among the predicted GO terms, there may be functionally related ones or those with hierarchical relationships such as ancestors and offsprings. These relationships introduce redundancy in functional annotations. It is challenging to define a specific gene count threshold in enrichment analysis in order to balance redundancy removal and functional completeness. To address this issue, we calculate similarity scores for clustering and employ word cloud statistics to achieve a tradeoff between minimizing redundancy and preserving the completeness of functional annotations.

In summary, by leveraging the transfer graph learning strategy, LncADeep 2.0 demonstrates its effectiveness in predicting: (i) general functions of uncharacterized lncRNAs in both physiological and pathological processes; (ii) specific functions within RNA-seq data under specific tissue or cell-type conditions. As more latent functions and associated molecules are identified in the future, the combination of computational methods and experimental validation will undoubtedly offer valuable insights into lncRNA research.

## Supplementary Material

btag162_Supplementary_Data

## Data Availability

The software implementation and documentation are available at https://doi.org/10.5281/zenodo.17164767. It is also provided at the Github repository https://github.com/Jefferson-Chou/LncADeep2. The transcripts utilized for training and evaluating the identification module were retrieved from GENCODE (https://www.gencodegenes.org/human/release_45.html). The original network interaction data were derived from the following databases: ENCORI (https://rnasysu.com/encori/index.php), STRING (https://cn.string-db.org), and RNAInter (http://www.rnainter.org/). Bulk RNA-seq data from the brain cortex, spleen, and testis were obtained from the GTEx Analysis V10 release (https://www.gtexportal.org/home/downloads/adult-gtex/bulk_tissue_expression).
