# Supplementary material for: A novel deep learning-driven framework for improving lncRNA comprehensive annotation with LncADeep 2.0
Source: Bioinformatics. 2026 Apr 1;42(4):btag162. doi: 10.1093/bioinformatics/btag162 (PMC13090826; doi:10.1093/bioinformatics/btag162)
Supplement: btag162_Supplementary_Data [file btag162_supplementary_data.zip › Supplementary materials.pdf]

Content:

1. Supplementary methods

1.1 Data preprocessing for the target task of the transfer learning strategy.

1.2 The parameters of GO enrichment.

1.3 Visualization of GO enrichment analysis.

1.4 Evaluation of functional annotation module of LncADeep 2.0.

2. Supplementary figures and legends

3. References

Supplementary methods

1.1 Data preprocessing for the target task of the transfer learning strategy

With the aim of predicting function-related proteins of lncRNAs, 12,650 lncRNAs in the interacting network were retrieved from the Ensembl database. As a result, 207 lncRNAs were annotated with 54 GO terms, encompassing 17,196 protein-coding genes (PCGs). Among these 54 GO terms, 30 were assigned to biological processes (BP), 13 to cellular components (CC), and 11 to molecular functions (MF). In this study, we focused on BP due to their closer association with lncRNA function. Since GO terms were organized hierarchically, with many child terms, we filtered out GO terms that have over 15 child terms to refine our analysis (Supplementary Fig. S1). On one hand, a higher number of child terms indicated that the GO term resided higher in the hierarchy and possessed a more general meaning, making it less relevant to our specific objectives. On the other hand, GO terms with more child terms typically encompassed a greater number of proteins, which hindered the prediction of function-related proteins of lncRNAs. After filtering, 110 lncRNAs and 21 GO terms (BP), including 1,724 proteins, were retained.

1.2 The parameters of GO enrichment

In the GO enrichment analysis, the option maxGSSize controlled the maximal size of genes annotated by ontology terms. The filtering of predicted function-related proteins further enhanced the reliability of the result. To this end, we set a series of gradients for adjustment of the maxGSSize parameter and the proportion of function-related protein filtering in the enrichment analysis. For example, as for the option maxGSSize, “2500 & 200” meant that when maxGSSize was set to 2500, the top 10 GO terms were selected from each of the following two conditions: (1) GO terms with a gene number $\leqslant$ 200, and (2) GO terms with a gene number between 200 and 2500. These two sets of top 10 GO terms were then combined. We then tested these parameters using the lncRNAs from the training set. The top 20 GO terms from the

functional annotations of the lncRNAs in the training set were used to evaluate the result: (1) the numbers (n) and ranks of exact matches with the labels in the training set, and (2) the semantic similarity of the labels in the training set. We ultimately chose maxGSSize as "2500 & 200", with the proportion of function-related proteins filtered set to 1/3, i.e., removing the bottom 2/3 of proteins based on prediction probability (Supplementary Fig. S2).

1.3 Visualization of GO enrichment analysis

To present the results of GO enrichment analysis more concisely, we calculated the similarity between different terms using Jaccard (JC) similarity and employed the Ward's method to elucidate the relationships among the annotated terms. Based on clustering results, the treeplot function in R package enrichplot was utilized to visualize the lncRNA functional annotation. The GO terms in the results were ranked by the adjusted p-values. The JC similarity and the distance could be described as

$$\mathrm{JC}(\mathrm{A},\mathrm{B}) = \frac{|\mathrm{A} \cap \mathrm{B}|}{|\mathrm{A} \cup \mathrm{B}|}, \tag{1}$$

$$\mathrm{Dist}(\mathrm{A},\mathrm{B}) = 1 - \mathrm{JC}(\mathrm{A},\mathrm{B}). \tag{2}$$

Where A, B represented the sets of genes annotated in GO terms A and B respectively. The distance matrix was derived by subtracting the similarity matrix from one.

1.4 Evaluation of functional annotation module of LncADeep 2.0

To evaluate the performance of predicting function-related proteins of lncRNAs, we conducted a stringent 5-fold test by partitioning the paired dataset of 110 lncRNAs and 1,724 BP-related proteins, ensuring that there were no overlapping proteins between the training and test parts (functional annotation evaluation data A). To figure out whether negative transfer occurred during transfer learning, we performed another split on the paired dataset, where the training and test parts contained non-overlapping

lncRNAs (functional annotation evaluation data B). For the above-mentioned evaluation data, we trained the model using the training part and predicted the functional correlations between lncRNAs and proteins in the test part.

Besides, we evaluated the performance of GO annotation prediction for lncRNAs. Given that distinct GO terms could represent similar biological functions, the semantic similarity was assessed between the predicted GO terms and the reference annotations for each lncRNA. The semantic similarity was calculated with the R package GOSemSim [1] by the Jiang and Conrath's method [2], which was information content-based. The information content (IC) of a GO term t was defined as follows:

$$IC(t) = -\log(p(t)), \tag{3}$$

$$p(t) = \frac{n_c}{N}, \tag{4}$$

where n_c was the number of children terms of GO term t, and N was the total number of terms in GO corpus.

IC-based methods considered the information contents of two GO terms' closest common ancestor term, also called most informative common ancestor (MICA). The Jiang's similarity could be computed by:

$$Sim_{Jiang}(t1, t2) = 1 - \min[1, IC(t1) + IC(t2) - 2IC(MICA)]. \tag{5}$$

The pairwise similarity was calculated between predicted GO terms and the ground truth. For each predicted term, we took the highest similarity. Regarding the similarity threshold, we adopted 0.5 as the cutoff value, which was commonly used as reported by Bettembourg et al [3]. We followed this evaluation process on the LncRNA2GO-68 dataset.

Supplementary figures

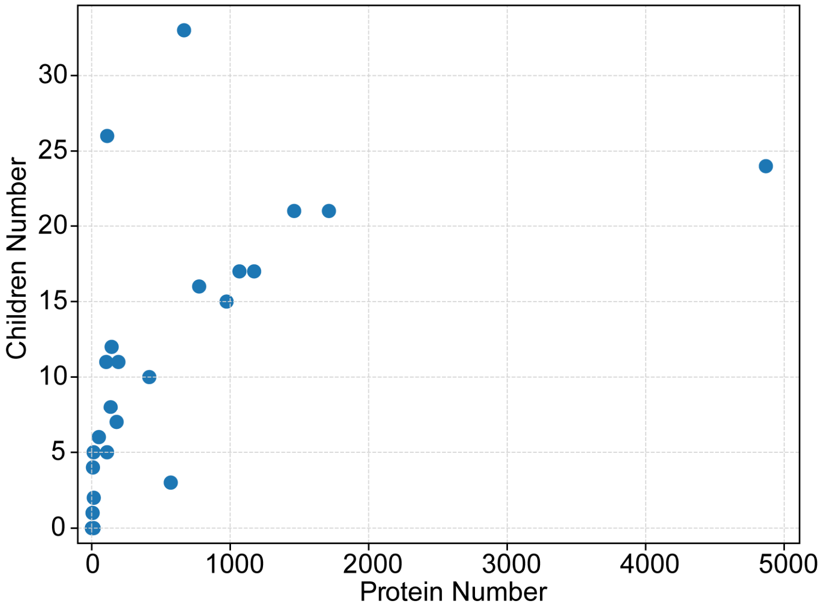

Supplementary Figure 1. Numbers of proteins and child terms of GO terms (BP).

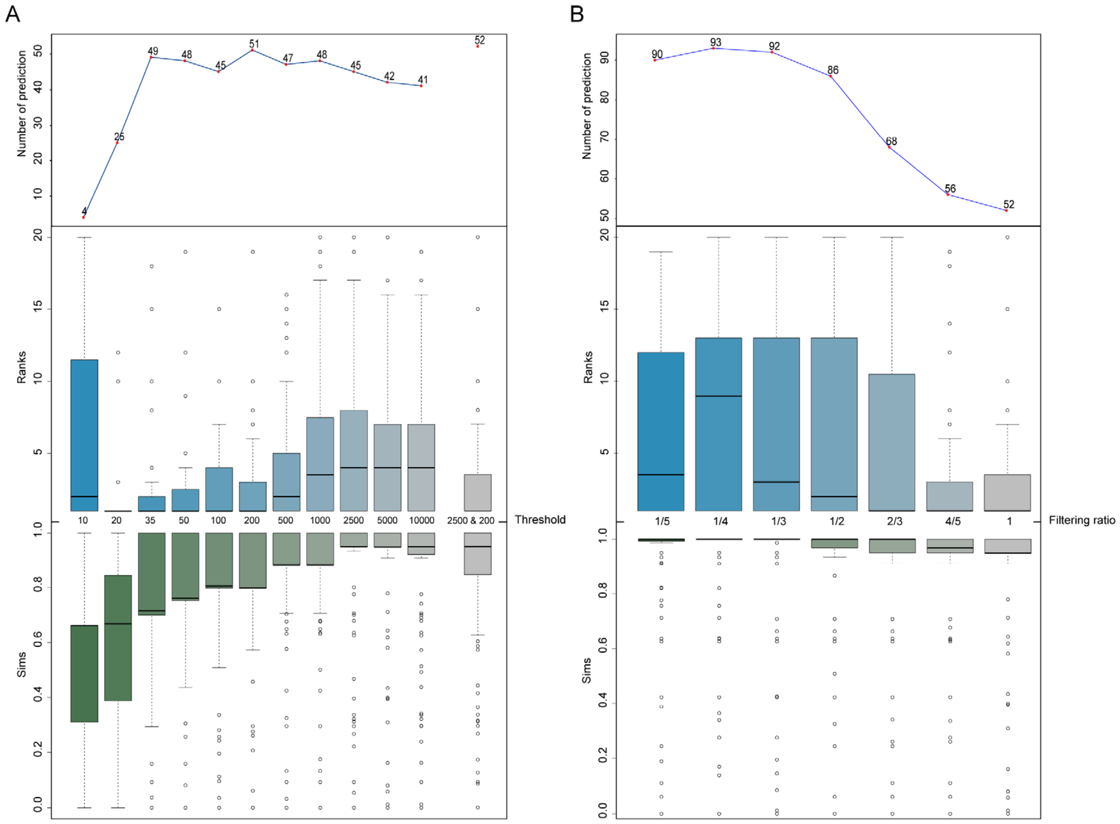

Supplementary Figure 2. Determination of filtering ratio of function-related proteins and thresholds of maxGSSize option. (A) Under different maxGSSize parameter settings, the number and ranking distribution of predicted GO terms that exactly matched the labels in the training set; and the semantic similarity distribution between the predicted GO terms and the training set labels for the lncRNAs. (B) Under different filtering ratios of function-related proteins, the number and ranking distribution of predicted GO terms that exactly matched the labels in the training set; and the semantic similarity distribution between the predicted GO terms and the training set labels for the lncRNAs.

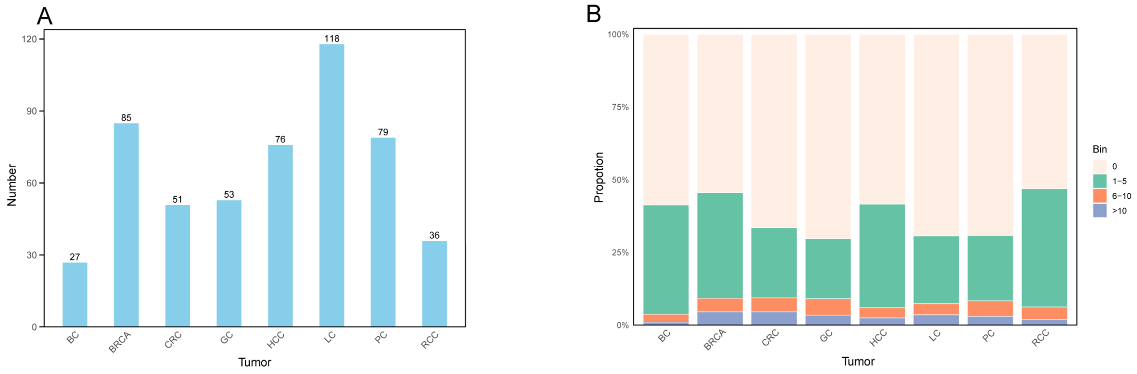

Supplementary Figure 3. LncADeep 2.0 infers tumor-association for lncRNAs by predicting relation between tumor markers and lncRNAs. (A) The number of lncRNAs related to ⩾ 15 markers absent from the Lnc2Cancer 3.0 database. (B) The number of function-related markers of 12,650 lncRNAs.

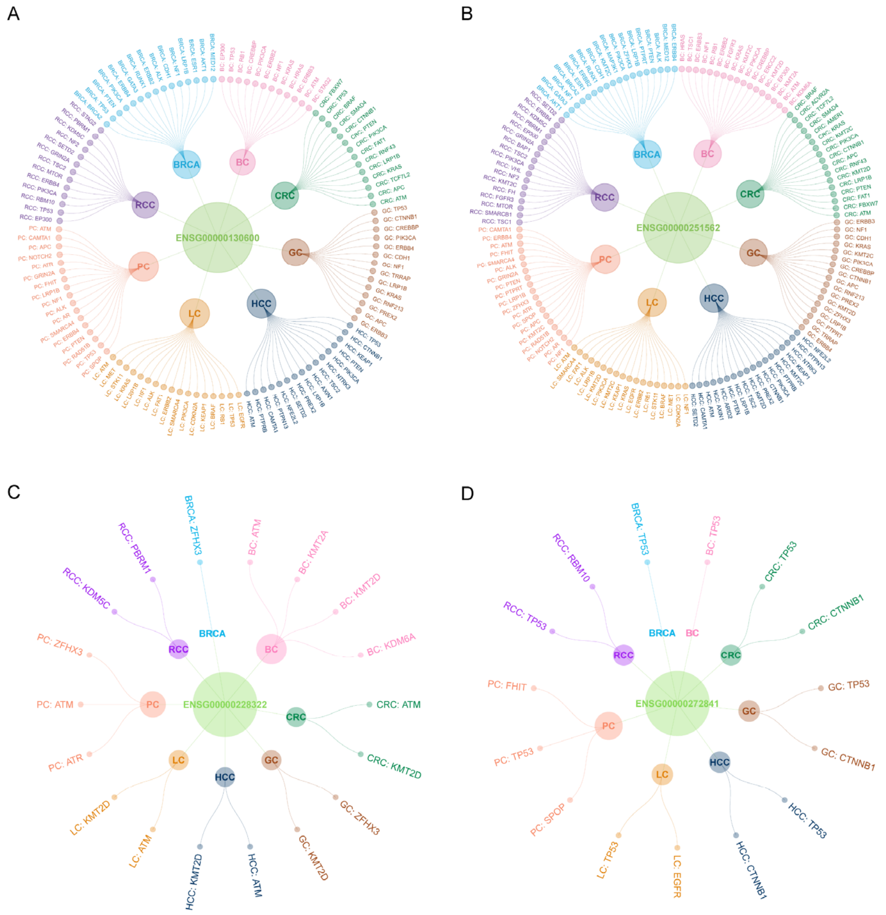

Supplementary Figure 4. Display of lncRNA-tumor-marker networks. The lncRNA-tumor-marker networks of *H19* (A), *MALAT1* (B), *GLIS3-AS2* (C) and *MAP3K4-AS1*(D). The size of a tumor circle represents the number of markers predicted to be correlated to the target lncRNA. The widely reported tumor-associated lncRNAs *H19* and *MALAT1* exhibit denser connections with a greater number of tumor markers within the network.

References

1. Yu G, Li F, Qin Y, et al. GOSemSim: an R package for measuring semantic similarity among GO terms and gene products. *Bioinformatics* 2010;**26**:976–978.
2. Jiang J, Conrath DW. Semantic Similarity Based on Corpus Statistics and Lexical Taxonomy. In: Proceedings of the 10th Research on Computational Linguistics International Conference. Taipei, Taiwan: ACLCLP. 1997.
3. Bettembourg C, Diot C, Dameron O. Optimal Threshold Determination for Interpreting Semantic Similarity and Particularity: Application to the Comparison of Gene Sets and Metabolic Pathways Using GO and ChEBI. *PLoS ONE* 2015;**10**:e0133579.
